# Top Differential Diagnosis Should Be Microscopic Polyangiitis in ANCA-Positive Patient with Diffuse Pulmonary Hemorrhage and Hemosiderosis

**DOI:** 10.1155/2014/286030

**Published:** 2014-11-30

**Authors:** Nicholas D. Ward, Diane E. Cosner, Colleen A. Lamb, Wei Li, Jacqueline K. Macknis, Michele T. Rooney, Ping L. Zhang

**Affiliations:** Department of Anatomic Pathology, William Beaumont Hospital, 3601 W 13 Mile Road, Royal Oak, MI 48073, USA

## Abstract

A rat model of antineutrophil cytoplasmic antibody (ANCA) associated vasculitides reveals crescentic glomerulonephritis as seen in human renal biopsies and diffuse lung hemorrhage that is not well documented in human lung biopsies. A 64-year-old male, with shortness of breath and mild elevation of serum creatinine, was found to have a positive serum test for ANCA, but negative antiglomerular basement membrane antibody. A renal biopsy showed pauci-immune type of crescentic glomerulonephritis and focal arteritis. The prior lung wedge biopsy was retrospectively reviewed to show diffuse hemorrhage and hemosiderosis with focal giant cells. In addition, small arteries revealed subtle neutrophil aggregation, and margination along vascular endothelium, but no definitive vasculitis. The pathology of ANCA associated vasculitides results from activated neutrophils by ANCA and subsequent activation of the alternative complement cascade with endothelial injury, neutrophil aggregation and margination. Our findings, after the correlation between lung biopsy and renal biopsy, imply that the top differential diagnosis in the lung biopsy should be microscopic polyangiitis when diffuse pulmonary hemorrhage and hemosiderosis are present in this ANCA-positive patient.

## 1. Introduction

In humans, ANCA associated systemic vasculitides clinically range from microscopic polyangiitis to granulomatosis with polyangiitis (used to be called Wegner's granulomatosis) to eosinophilic granulomatosis with polyangiitis (used to be called Churge-Strauss syndrome). The systemic vasculitides mainly involve small vessels of the lungs and kidneys, causing compromised lung function and rapid progressive renal failure [1–4]. Conventionally, lung biopsies are characterized by necrotizing granulomatous inflammation and vasculitis as seen in granulomatosis with polyangiitis [5–7] and eosinophilic granulomatosis with polyangiitis contain more eosinophils in vasculitis in addition to changes seen in granulomatosis with polyangiitis. However, pulmonary hemorrhage and hemosiderosis as a manifestation of ANCA positive microscopic polyangiitis have not been well established [8, 9]. In patients with positive ANCA, renal biopsies mainly show crescentic formation in glomeruli as one unique feature of vasculitis (called primary crescentic glomerulonephritis), leading to acute renal failure [4, 10]. Based on two animal models (one in mice and the other in rats) with ANCA associated CGN, lymphocytes are activated to differentiate into plasma cells for producing a circulating ANCA antibody [2, 11–14]. ANCA then activates neutrophils via binding ANCA antigen in the neutrophils, leading to vasculitis through an interaction with complement activation, and stimulates crescent formation from proliferative parietal epithelium. The rat model of ANCA associated vasculitides reveals crescentic glomerulonephritis as seen in human renal biopsies and diffuse lung hemorrhage [11] that is not well documented in human lung biopsies. We correlated both the lung biopsy and renal biopsy in a patient with positive ANCA and suggest that microscopic polyarteritis should be in the top differential diagnosis for findings of diffuse hemorrhage and hemosiderosis in the lung under this condition.

## 2. Case Report

**Figure 1:**
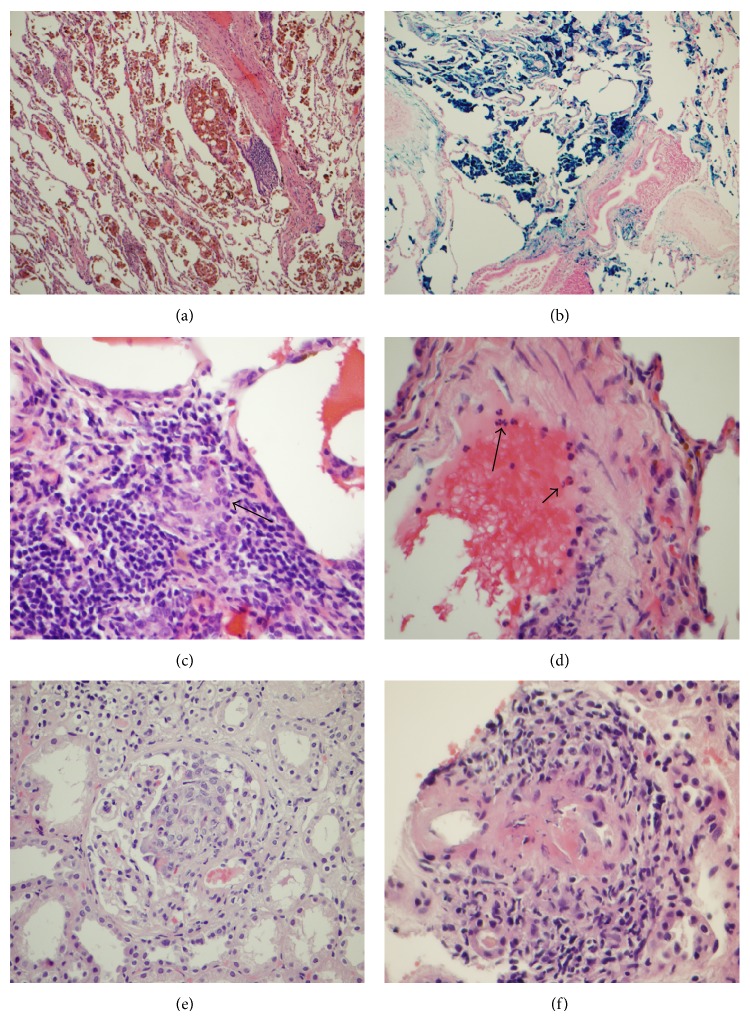
ANCA associated vasculitides in a 64-year-old man. In the wedge lung biopsy, there was diffuse alveolar hemorrhage (a). The hemosiderosis (hemosiderin-laden macrophages) was confirmed by positive iron staining in (b) (magnification ×100 for both (a) and (b)). High power view in the lung showed focal giant cell (c) and neutrophil aggregate and margination along the endothelium of an artery (d). Renal biopsy revealed cellular crescent formation (e) and focal fibrinoid arteritis (f). Magnification ×400 in (c)–(f).

A 64-year-old male had progressive dyspnea over six-month duration. The patient had a history of asthma but never smoked. A recent cardiac evaluation included normal echocardiogram and stress test. Computerized tomography (CT) chest imaging without contrast revealed bilateral mild ground-glass opacities in the lungs, slightly greater within the middle and lower lobes on the right side, suggestive of mild interstitial lung disease. He was found to have positive serum p-ANCA at titer of 1 : 640 while the antiglomerular basement membrane antibody was negative. His serum test for myeloperoxidase was positive at greater than 8 units (normal < 0.4 unit) while his serum level of proteinase-3 was negative. Wedge lung biopsies from the right upper, middle, and lower lobes were performed to reveal red to purple color from pleural surface and red color on the cross sections grossly. Microscopically, the biopsies mainly revealed diffuse hemorrhage and hemosiderosis (iron positive macrophages in alveoli) (Figures 1(a) and 1(b)), but no definite vasculitis was present. The case was sent for expert opinion with a diagnosis of idiopathic pulmonary hemosiderosis. They commented that (1) findings were consistent with a diffuse alveolar hemorrhage syndrome; (2) the absence of vasculitis and capillaritis argued against the most common pulmonary vasculitides, namely, granulomatosis with polyangiitis, eosinophilic granulomatosis with polyangiitis, and microscopic polyangiitis; and (3) Goodpasture's syndrome was possible but required the identification of antiglomerular basement membrane antibodies. One month later, because the lung biopsies were not conclusive and the patient showed repeated positivity for ANCA while a mild elevation of serum creatinine (1.23 mg/dL) and mild hematuria was present, then a renal biopsy was done to rule out ANCA associated crescentic glomerulonephritis.

The patient's renal biopsy showed cellular crescents in 3 of 12 glomeruli and focal fibrinoid vasculitides in a small artery (Figures 1(e) and 1(f)) with mild interstitial fibrosis. Immunofluorescent stains for IgG, IgA, IgM, C1q, kappa, and lambda were all negative in the glomeruli, except trace nonspecific C3 staining in mesangial areas, and electron microscopy did not demonstrate either immune complex deposits or fibril materials. With the given positive serology for ANCA, the overall findings in the renal biopsy were consistent ANCA associated pauci-immune type of crescentic glomerulonephritis and vasculitis. Then the lung wedge biopsies were retrospectively reviewed to confirm diffuse hemorrhage and hemosiderosis but with focal giant cells (Figure 1(c)). In addition, small arteries revealed subtle neutrophil aggregation and margination along vascular endothelium (Figure 1(d)). Retrospectively, we felt that diffuse hemorrhage and hemosiderosis in the lung were most likely associated ANCA related vasculitis, namely, microscopic polyangiitis, in absence of traditional morphology of either necrotizing granulomatous inflammation or fibrinoid vasculitis in the lung. Our retrospective interpretation of microscopic polyangiitis in the lung was further supported by the previously mentioned consulting institute later on.

## 3. Discussion

**Figure 2:**
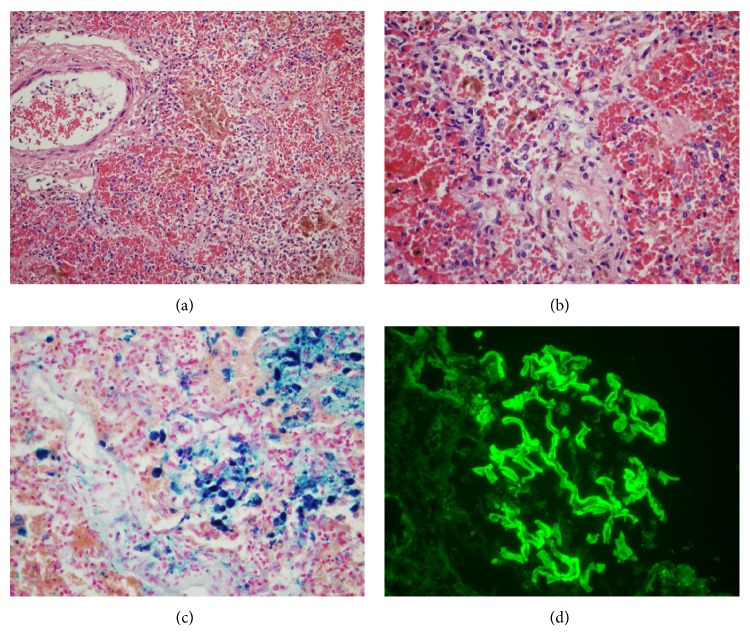
Goodpasture's syndrome in a 26-year-old woman. The patient presented with rapid progressive dyspnea over 2 weeks. Twice bronchoalveolar lavages showed hemosiderin-laden macrophages in the cytology specimens. Later she was found to have positive serum level for antiglomerular basement membrane antibody, although her renal function was not obviously compromised. The patient expired despite intensive care. Microscopically, there was diffuse alveolar hemorrhage and hemosiderosis in the bilateral lung sections at the autopsy (panel (a), ×100, and panel (b), ×400). The hemosiderosis was confirmed by diffuse positive iron staining in hemosiderin-laden macrophages in panel (c) ((c), ×400). In panel (d), glomeruli were positive for linear IgG staining on immunofluorescent section, confirming antiglomerular basement membrane disease ((d), ×400), but no crescent formation was identified in the glomeruli. Overall autopsy findings were consistent with Goodpasture's syndrome with dominant pathologic changes in her lungs.

Pulmonary hemosiderosis indicates chronic hemorrhage, as iron positivity is identified in hemosiderin-laden macrophages. Traditionally, the differential diagnosis for diffuse pulmonary hemosiderosis includes idiopathic pulmonary hemosiderosis versus Goodpasture's syndrome, which is supported by a positive test for antibasement membrane antibody [15, 16]. Goodpasture's syndrome affects children more commonly than adults. We had a recent case in a young woman with fatal Goodpasture's syndrome showing diffuse pulmonary hemorrhage and hemosiderosis (Figure 2). When the glomerular capillary loops are the target vascular bed and alveolar capillaries are terminal targets for the attack of antibasement membrane antibodies, primary crescentic glomerulonephritis and alveolitis with pulmonary hemorrhage are expected to be seen in Goodpasture's syndrome. Because ANCA associated crescentic glomerulonephritis is another primary crescentic glomerulonephritis, alveolar capillaries and small arteries can be certainly the targets for ANCA associated vasculitis as well; thus, a differential diagnosis of pulmonary hemosiderosis and diffuse alveolar bleeding should include ANCA associated microscopic polyangiitis in patients with positive ANCA. Traditional criteria of ANCA associated vasculitis including necrotizing granulomatosis and vasculitis were initiated as early as 1930 [4], whereas ANCA was not discovered until 1982 [17]. In experienced hands, there is more than 90% correlation between positive ANCA and identification of pauci-immune crescentic glomerulonephritis. Nephropathologists have used positive tests of ANCA to correlate with pauci-immune crescentic glomerulonephritis for more than 2 decades [10]; thus, the patients with ANCA associated crescentic glomerulonephritis can be adequately treated [18, 19]. Furthermore, ANCA associated vasculitides are characterized by diffuse hemorrhage in the lungs and crescentic glomerulonephritis in the kidneys in a rat model [11, 14]. Finally, as activated neutrophils are found to trigger the activation of complement cascade and subsequent endothelialitis [4, 12, 13], neutrophil aggregation and margination should be taken as a feature of early vasculitis. In fact, neutrophil margination and pericapillary neutrophils have been used for assisting antibody mediated rejection in renal transplant biopsies for years [20, 21].

Unlike Goodpasture's syndrome with one positive antibody and one main finding of pulmonary hemorrhage and hemosiderosis, ANCA associated vasculitis can range from microscopic polyangiitis to granulomatosis with angiitis and eosinophilic granulomatosis with polyangiitis in lung biopsies. Pathologic morphology of ANCA associated microscopic polyangiitis in lung biopsies is not well established [8, 9], but scattered reports regarding alveolitis and hemoptysis as respective pathologic finding and clinical symptom exist [22–24]. Even granulomatosis with angiitis can be present with diffuse lung hemorrhage [25]. The deceiving factor in our current case with microscopic polyangiitis of lung and the Goodpasture's syndrome presented in Figure 2 was the fact that renal function appeared relatively normal in both patients during the early course of their disease. We feel there is a definite need in the pulmonary pathology field to study whether pulmonary hemorrhage and hemosiderosis with neutrophil margination as features of microscopic polyangiitis can be correlated with positive serum tests of ANCA. In addition, other serum markers such as antiglomerular basement membrane antibody (20% change overlapping with positive ANCA [26]) and angiotensin converting enzyme for sarcoidosis [27] should be tested before any invasive lung biopsies to rule out infection, interstitial lung disease with unknown etiology, and vasculitis/granulomatosis/sarcoidosis. In our current case report, we found a triangle correlation among positive ANCA test, pulmonary hemorrhage and hemosiderosis with neutrophil margination as signs of a subtle microscopic polyangiitis, and pauci-immune crescentic glomerulonephritis/vasculitis in the renal biopsy.

In summary, a positive ANCA test is an additional serology biomarker suggestive of systemic vasculitis. The criteria for ANCA associated variants of pulmonary vasculitis should be further investigated beyond traditional morphologic findings, which were established many years before ANCA test was discovered. The animal model of ANCA associated systemic vasculitides appears coupled with our findings after the current correlation between the lung biopsy and renal biopsy, implying that microscopic polyangiitis should be on the top differential diagnosis when diffuse pulmonary hemorrhage and hemosiderosis are the main findings in ANCA-positive patients.
